# Evaluation of *Gaussia* luciferase and foot-and-mouth disease virus 2A translational interrupter chimeras as polycistronic reporters for transgene expression

**DOI:** 10.1186/s12896-017-0367-0

**Published:** 2017-06-12

**Authors:** Michael Puckette, Thomas Burrage, John G. Neilan, Max Rasmussen

**Affiliations:** 1 U.S. Department of Homeland Security Science and Technology Directorate, Plum Island Animal Disease Center, P.O. Box 848, Greenport, NY 11944 USA; 2 Oak Ridge Institute for Science and Education, Plum Island Animal Disease Center Research Participation Program (PIADC), P.O. Box 117, Oak Ridge, 37831 TN USA

**Keywords:** *Gaussia* luciferase, Foot-and-mouth disease virus, 2A, Bicistronic, Polycistronic, Biomarker, Virus-like particles

## Abstract

**Background:**

The *Gaussia princeps* luciferase is used as a stand-alone reporter of transgene expression for in vitro and in vivo expression systems due to the rapid and easy monitoring of luciferase activity. We sought to simultaneously quantitate production of other recombinant proteins by transcriptionally linking the *Gaussia princeps* luciferase gene to other genes of interest through the foot-and-mouth disease virus 2A translational interrupter sequence.

**Results:**

We produced six plasmids, each encoding a single open reading frame, with the foot-and-mouth disease virus 2A sequence placed either N-terminal or C-terminal to the *Gaussia princeps* luciferase gene. Two plasmids included novel *Gaussia princeps* luciferase variants with the position 1 methionine deleted. Placing a foot-and-mouth disease virus 2A translational interrupter sequence on either the N- or C-terminus of the *Gaussia princeps* luciferase gene did not prevent the secretion or luminescence of resulting chimeric luciferase proteins. We also measured the ability of another polycistronic plasmid vector with a 2A-luciferase sequence placed downstream of the foot-and-mouth disease virus P1 and 3C protease genes to produce of foot-and-mouth disease virus-like particles and luciferase activity from transfected cells. Incorporation of the 2A-luciferase sequence into a transgene encoding foot-and-mouth disease virus structural proteins retained luciferase activity and the ability to form virus-like particles.

**Conclusions:**

We demonstrated a mechanism for the near real-time, sequential, non-destructive quantitative monitoring of transcriptionally-linked recombinant proteins and a valuable method for monitoring transgene expression in recombinant vaccine constructs.

## Background

Real-time sequential monitoring of recombinant protein production is advantageous over single-event, terminal monitoring that requires destruction of expressing cells in vitro or the *ex vivo* analysis of clinical samples. For example, transfected cell cultures may require lysis for detection of recombinant proteins of interest through polyacrylamide gel electrophoresis, western blots, ELISA, indirect fluorescent antibody assay or other methods. These detection methods are time-consuming, costly and often require protein-specific antibody reagents. Monitoring in vivo expression of recombinant proteins is more problematic. It requires invasive sampling at fewer time points, or terminal procedures, as well as protein-specific reagents.

Linking expression of a recombinant protein of interest to an easily detectable, secreted biomarker in a single open reading frame would allow for rapid, quantitative, and sequential monitoring of all proteins within the transcriptional unit. Moreover, using a secreted biomarker would be a useful tool for quantitating in vivo recombinant protein expression independent from host immune responses.

The *Gaussia princeps* luciferase (GLuc) is a naturally secreted luciferase that catalyzes oxidation of the substrate coelenterazine to produce an intense luminescent burst [1, 2]. GLuc is readily quantifiable in clinical samples (i.e. blood, plasma, and urine) within a linear detection range [3–8]. The luminescent output of wild-type GLuc is enhanced by mutation of two amino acid residues, F89W and I90L, resulting in a super-luminescent GLuc variant (SGLuc) with a peak emission wavelength of 481 nm [9]. We sought to use secreted GLuc (and GLuc variants) as a general biomarker to monitor overall expression of recombinant proteins from a single transcriptional unit. GLuc is non-native to the mammalian system. This allows for more definitive quantification than other enzymatic biomarkers, such as secreted embryonic alkaline phosphatase, which can have innate levels in vivo [10].

Production of multiple recombinant proteins from a single open-reading frame has been previously accomplished through use of proteolytic cleavage, self-processing peptides, multiple internal ribosome entry sites (IRESs), and other mechanisms [11, 12]. Foot-and-mouth disease virus (FMDV) encodes a nonstructural 2A translational interrupter which induces ribosome skipping causing the separation of the FMDV P1 and P2 polyproteins in a non-proteolytic manner [13, 14]. The efficiency of FMDV 2A-mediated translational interruption is amino acid sequence dependent, and its activity is enhanced when the additional sequence derived from the C-terminus of the FMDV 1D (VP1) protein is included [15, 16]. FMDV 2A-mediated polyprotein separation is nearly 100% efficient and produces a constant 1:1 yield of proteins on either side of the FMDV 2A sequence [14]. Therefore, a fusion of GLuc and FMDV 2A sequences potentially provides a mechanism to directly correlate yields of transcriptionally-linked recombinant proteins by assaying for secreted GLuc activity. Such an assay would enable sequential, non-destructive sampling and normalization among test samples.

We report the production and evaluation of six distinct chimeras of GLuc or SGLuc (GLuc/SGLuc) variants with the FMDV 2A translational interrupter on either the N- or C-terminus within a single open reading frame, including two novel GLuc/SGLuc variants with a deleted methionine start codon. We also evaluated the ability of one chimera to function as the 3′ terminus of a transgene encoding a FMDV P1-2A-3C cassette known to produce VLPs.

## Results

### Design of six bicistronic GLuc/SGLuc constructs

**Fig. 1:**
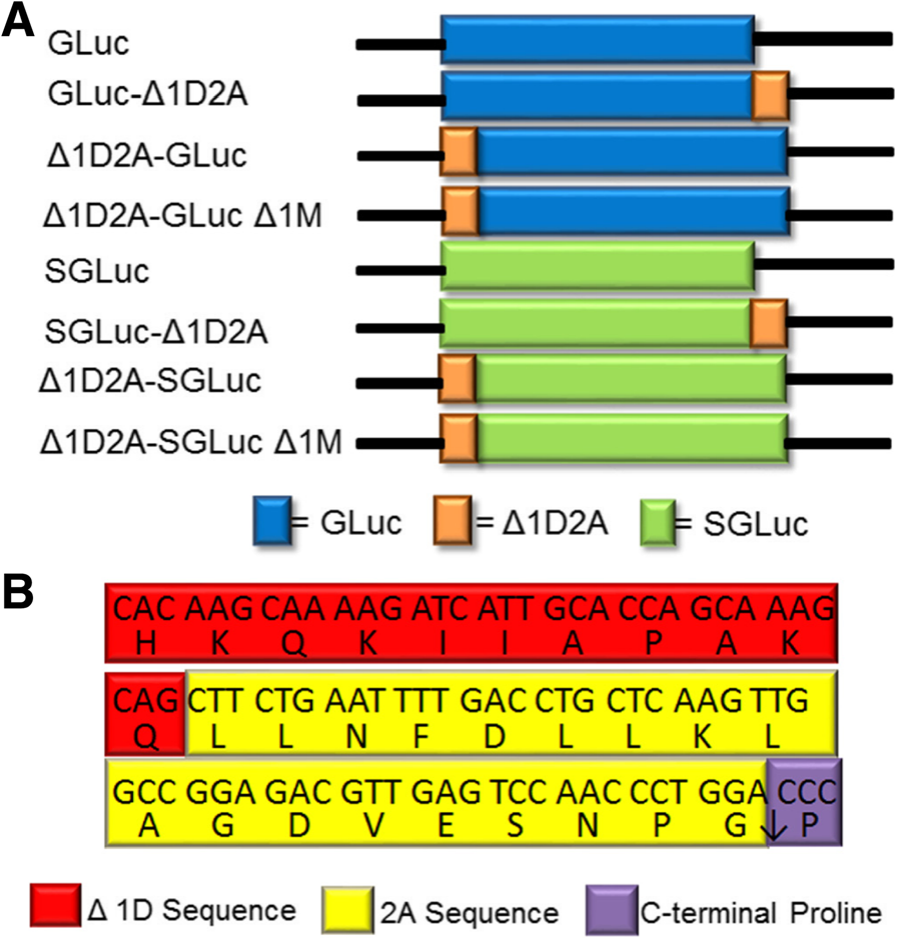
**a** Construct layouts of bicistronic templates evaluated. **b** Nucleotide and amino acid sequences of Δ1D2A translational interrupter

**Fig. 2:**
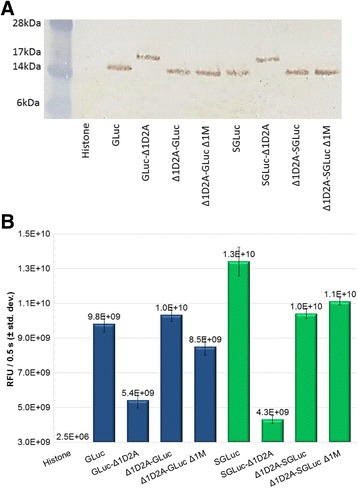
**a** Western blot probed with Anti-GLuc antibody showing roughly equalized loading. **b** Cell culture media luciferase readings for bicistronic templates using volumes determined by western blotting. RFUs, relative fluorescent units

A total of six bicistronic GLuc/SGLuc constructs were evaluated for retention of secretion and ability to luminesce (Fig. 1a). To facilitate efficient translational interruption in bicistronic constructs, we used a modified FMDV 2A sequence identified as Δ1D2A [17] consisting of the 11 C-terminal amino acids of VP1 (Δ1D), and the defined 18 amino acid 2A sequence with a C-terminal proline (Fig. 1b) [16]. Four bicistronic constructs had the Δ1D2A sequence inserted on either the N- or C-terminus of either GLuc/SGLuc. We found that the methionine normally at position 1 was dispensable for translation initiation when the Δ1D2A was positioned on the N-terminus of GLuc/SGLuc (Fig. 2, results discussed below). Consequently, the final two bicistronic constructs were composed of GLuc/SGLuc templates with an N-terminal Δ1D2A sequence and a deletion of the position 1 methionine within the luciferase (Δ1M) (Fig. 1a).

### Luciferase activity in cell culture

Addition of the C-terminal Δ1D2A sequence in GLuc-Δ1D2A and SGLuc-Δ1D2A constructs resulted in a molecular weight increase of resulting proteins as visualized in western blots used to establish relative sample loading prior to quantifying luciferase activities (Fig. 2a). Luciferase activity was present in all samples except the histone negative control confirming that addition of the Δ1D2A sequence on either the N- or C-terminus of the luciferase did not prevent secretion or luminescence (Fig. 2b).

Relative sample loading was used to account for the possibility that the addition of the Δ1D2A to GLuc/SGLuc may alter luminescent intensities. By establishing relative sample loading using an anti-GLuc polyclonal antibody we were able to observe that the GLuc-Δ1D2A construct displayed 45 and 48% reductions in luminescent output versus the unmodified GLuc and Δ1D2A-GLuc constructs (Fig. 2b). The SGLuc-Δ1D2A construct exhibited a 59 and 68% reduction relative to Δ1D2A-SGLuc and SGLuc constructs (Fig. 2b). Deletion of the GLuc/SGLuc start codon, Δ1M, from N-terminal chimeras did not prevent SGLuc secretion and resulted in an 18% reduction compared to Δ1D2A-GLuc Δ1M and a 6% increase when compared to Δ1D2A-SGLuc.

### Luciferase activity in cell lysis buffer

**Fig. 3:**
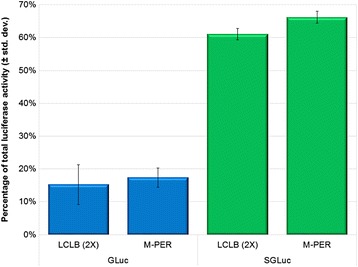
Percentage of total sample luciferase activity retained when mixed with one of two cell lysis buffers, LCLB (2×) or M-PER, as compared to cell culture media designated as 100%

Despite previous reports identifying the SGLuc variant as emitting approximately 10-fold higher luminescence than GLuc from lysed cells [9], we measured a 36% average increase in luminescence in cell culture media (Fig. 2b). To determine if the choice of cell lysis buffer affected the luminescence output between GLuc and SGLuc, we combined harvested cell media from each variant with one of two common cell lysis buffers (2× Luciferase Cell Lysis Buffer [LCLB] and Mammalian Protein Extraction Reagent [M-PER]), and measured luciferase levels (Fig. 3). The SGLuc and GLuc constructs lost >30 and >80% activity when diluted in either cell lysis buffer as compared to control cell culture media, but the SGLuc construct retained approximately four-fold more luciferase activity than the GLuc construct in either cell lysis buffer.

### A biomarker for expression in FMDV VLP producing constructs

**Fig. 4:**
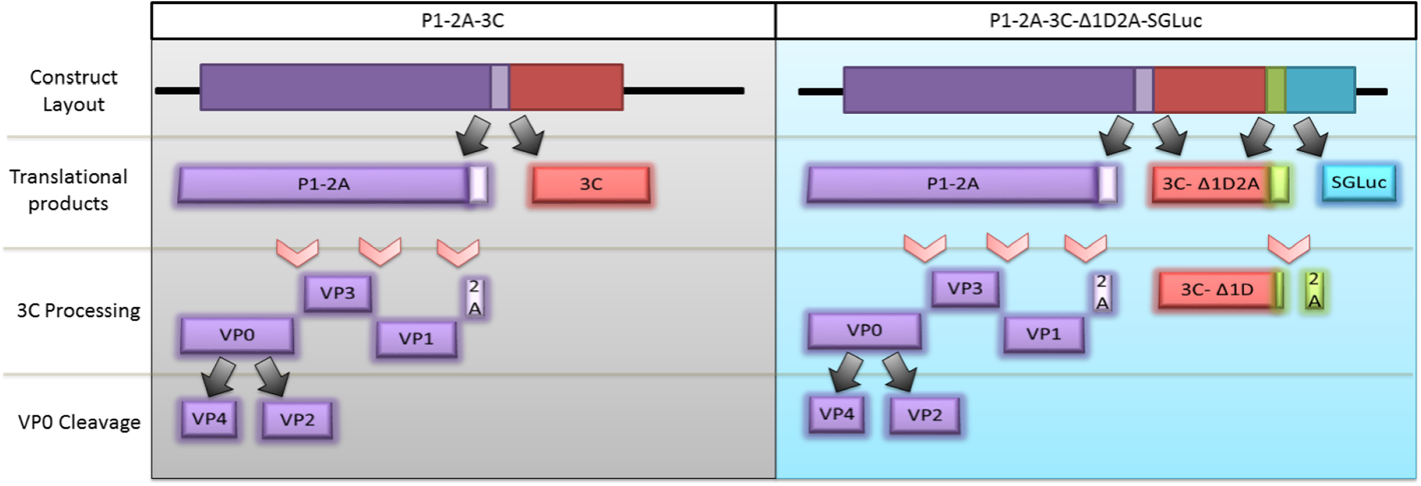
Construct layouts along with subsequently expressed and processed FMDV proteins used for the evaluation of VLP formation while utilizing the Δ1D2A-SGLuc sequence

**Fig. 5:**
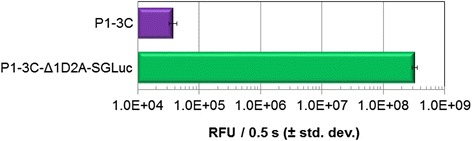
Luciferase activity from transfected LFBK-αVβ6 cell culture media. RFUs, relative fluorescent units

To determine if the transcriptionally-linked luciferase affected the ability of a FMDV transgene to produce virus-like particles (VLPs), we added the Δ1D2A-SGLuc chimera to the C-terminus of a plasmid vector, P1-2A-3C, capable of producing FMDV VLPs in transfected cell cultures (Fig. 4). Luciferase activity was readily detected in media from LFBK-αvβ6 cells transfected with the FMDV P1-2A-3C-Δ1D2A-SGLuc construct (Fig. 5).

**Fig. 6:**
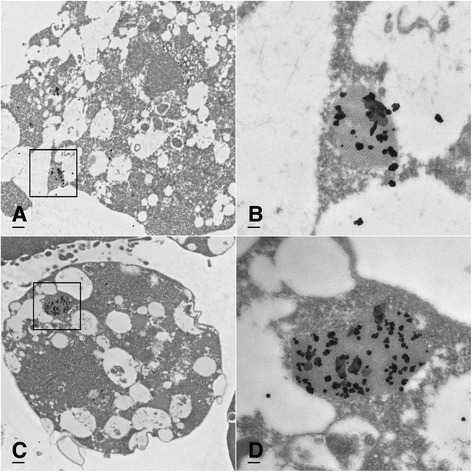
Immunoelectron microscopy images using immunogold coated with monoclonal antibody F21 to localize FMDV VP2 in the virus-like particle arrays of LFBK-αVβ6 cells transfected with construct P1-2A-3C-Δ1D2A-SGLuc at **a** 5000× (bar, 500 nm) and **b** 25,000× (bar, 100 nm) as well as construct P1-2A-3C at **c** 5000× (bar, 500 nm) and **d** 25,000× (bar, 100 nm)

**Fig. 7:**
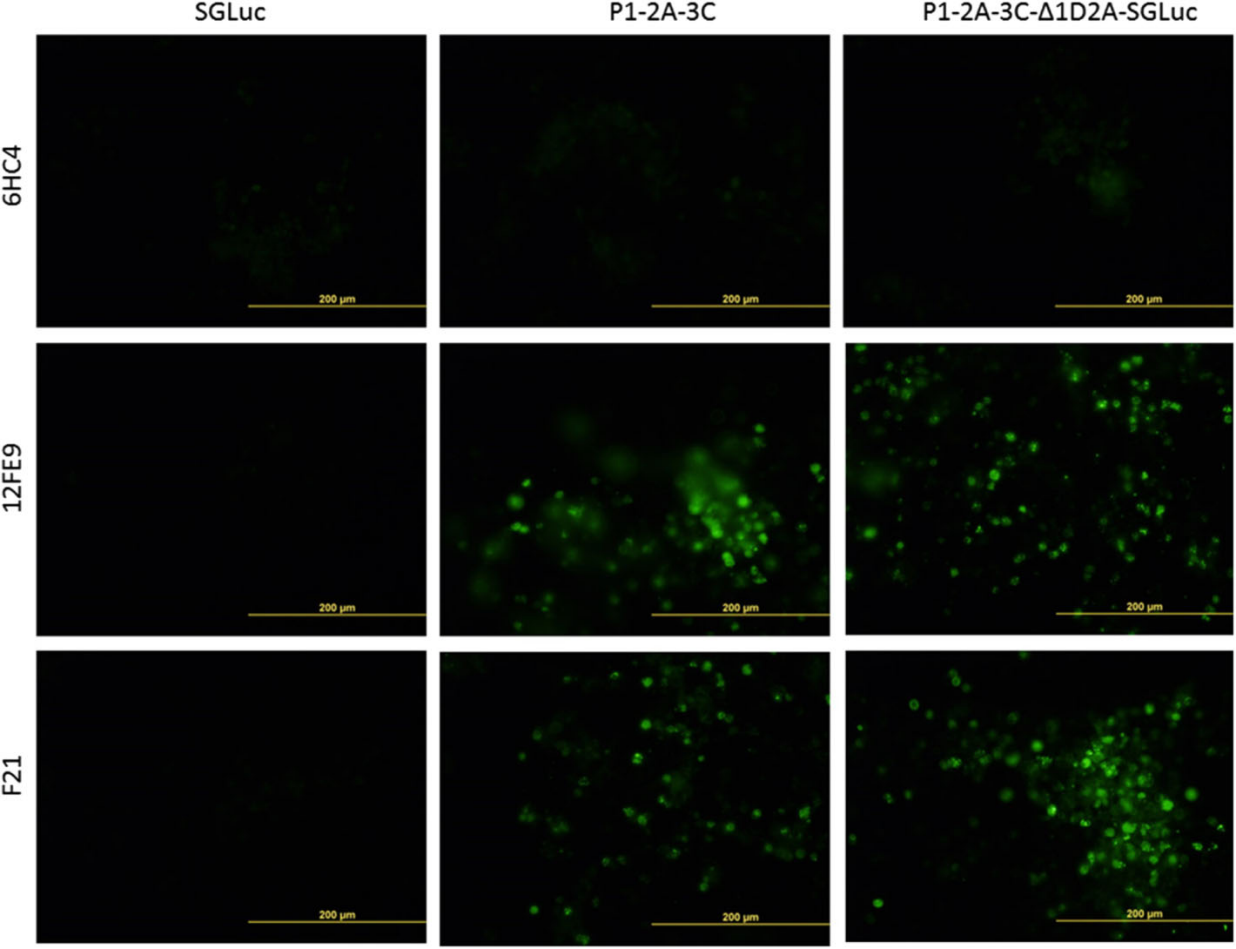
Immunofluorescent assay with FMDV specific monoclonal antibodies 6HC4, 12FE9 and F21 on LFBK-αVβ6 cells transfected with constructs encoding either SGLuc, P1-2A-3C, or P1-2A-3C-Δ1D2A-SGLuc (bar, 200 μm)

Using immunoelectron microscopy (IEM) we observed electron-lucent arrays of 25–30 nm diameter FMDV VLPs in the cytoplasm of LFBK-αvβ6 cells transfected with either P1-2A-3C or P1-2A-3C-Δ1D2A-SGLuc plasmids (Fig. 6). Immunogold labeled FMDV conformation dependent monoclonal antibody (Mab) (F21) localized to these array structures (Fig. 6). Immunofluorescent assays with two additional non-conformation dependent FMDV-specific Mabs, 6HC4 and 12FE9, further validated serotype-specific expression of FMDV proteins in transfected cells (Fig. 7).

## Discussion

We produced GLuc/SGLuc Δ1D2A luciferase chimeras that retained both secretory and luciferase activity, and demonstrated use of these chimeras as tools to quantitate transgene expression through sequential, non-destructive sampling of all culture media.

In our system, the SGLuc variant showed enhanced luminescence over GLuc, but at lower levels than previously reported [9]. We showed that this difference is influenced by the choice of cell lysis buffer, and may explain the differences in luciferase intensities observed between our results and those previously reported utilizing secretion-deficient mutants harvested from lysed cells [9]. The reduction of luciferase activity in cell lysis buffer may be due to the presence of denaturing conditions which may have more impact on GLuc than on SGLuc. Constructs containing a C-terminal Δ1D2A showed less luminescence despite having similar levels of protein tested, as determined by western blot. These data suggest that the luminescent activity of these chimeras was inhibited compared to N-terminal and GLuc/SGLuc constructs.

We produced a plasmid encoding the FMDV P1, 2A, 3C genes in-frame with the Δ1D2A-SGLuc sequence on the 3′ terminus. This marker-vaccine construct retained the ability to produce assembled FMDV VLPs along with a concomitant readout of luciferase activity in culture media as an easily quantifiable marker of FMDV protein production. Notably, the VLP crystalline arrays produced in this system appeared to be identical to those previously reported from cells infected with wildtype FMDV [18, 19]. The conformation dependent F21 Mab will only interact with VP2 in the proper confirmation, such as when it is contained in a VLP. As FMDV VLPs form and become localized within arrays it results in the localization of F21 binding to these arrays as observed in IEM.

Rapid quantitation of recombinant protein levels is a critical tool for molecular biology. The system described here provides a direct correlate to quantitate recombinant proteins produced in various matrices, including clinical samples, without the need for antigen-specific reagents. For development of recombinant vaccines, this system may provide a mechanism to monitor recombinant antigen production in vivo following vaccination, determine vaccine vector expression kinetics, optimize delivery methods, and correlate the amount of recombinant antigen produced with host immune response, and protection from infection. Lastly, this reporter system may be widely applicable to monitor transgene expression for a variety of biomedical purposes including gene therapy, drug screening, vaccine production, and bio-manufacturing.

## Conclusions

We describe the use of *Gaussia* luciferase chimeras as a reporter system for quantifying recombinant gene expression. GLuc/SGLuc variants with the FMDV Δ1D2A sequence placed on either the N- or C- terminus retained secretory and luciferase activity that enabled rapid, sequential quantitation of transgene expression through non-destructive sampling without need for protein-specific detection reagents. Placing the Δ1D2A-SGLuc sequence downstream of an FMDV VLP production cassette resulted in both VLP production and luciferase activity, thereby demonstrating the practical application of this technology to recombinant vaccine development.

## Methods

### Insertions into pTarget plasmid

GLuc template sequence was obtained from the pMCS-*Gaussia* Luc plasmid (Thermo Fisher Scientific). PCR was performed using OneTaq 2× Master Mix with Standard Buffer (New England Biolabs) and primers GLuc-F and GLuc-R (Table 1). Insertion into the pTarget vector (Promega) followed manufacturer’s instructions for T/A cloning. Plasmids were sequenced using primers T7 and Seq-R (Table 1), and analyzed using the Sequencher 4.8 program (Genecodes).

**Table 1:** Primers utilized in this study

Primer name	Sequence
GLuc-F	TTGGCGCGCCGCCACCATGGGAGTCAAA
GLuc-R	GAGGCTGATTTTGCGTCTAGA
T7	TAATACGACTCACTATAGGG
Seq-R	TTACGCCAAGTTATTTAGGTGACA
H3-F	AAGGAGCTCGAGCCACCATGGCTCGT
H3-R	GGTTACCTTAAGCACGTTCTCCA
SGLuc8990–MF	CCAAGATGAAGAAGTGGCTCCCAGGACGCTGCC
SGLuc8990–MR	GGCAGCGTCCTGGGAGCCACTTCTTCATCTTGG
GLuc-NS-NheI-R	CGGCGCTAGCGTCACCACCGGCCCCCTT
AscI-Kzk-GLuc-F	TTGGCGCGCCGCCACCATGGGAGTCAAA
2A-XmaI-R	TATACCCGGGTCCAGGGTTGGACTC
GLuc-R-NotI	GCGGCCGCTTAGTCACCACCGGCCCC
GLuc8990–MF	CCAAGATGAAGAAGTTCATCCCAGGACGCTGCC
GLuc8990–MR	GGCAGCGTCCTGGGATGAACTTCTTCATCTTGG
No Met GLuc-MF	TCCAACCCTGGGCCCGGAGTCAAAGTTCTG
No Met GLuc-MR	CAGAACTTTGACTCCGGGCCCAGGGTTGGA
NotI-3CLeb89-F	CAGCGGCCGCATGAGTGGTGCCCCACCG
3CLeb89-EcoRI-R	GAATTCCTACTCGTGGTGTGGTT
3CLeb89-ns-EcoRI-R	GAATTCCTCGTGGTGTGGTTC

PCR of bovine Histone H3 was performed using OneTaq 2× Master Mix with Standard Buffer (New England Biolabs) with primers H3-F and H3-R (Table 1), using cDNA synthesized from isolated bovine mRNA. Sequencing and analysis were performed as described above.

### Mutation to SGLuc

SGLuc was generated using the pTarget GLuc template and site-directed mutagenesis using the GENEART Site-Directed Mutagenesis System (Invitrogen) with primers SGLuc8990–MF and SGLuc8990–MR (Table 1). Sequencing confirmation utilized primers T7 and Seq-R (Table 1) as described above.

### Creation of GLuc/SGLuc-Δ1D2A chimeras

For creation of GLuc-Δ1D2A chimera, PCR was performed with pTarget GLuc as a template using OneTaq 2× Master Mix with Standard Buffer (New England Biolabs) and primers T7 and GLuc-NS-NheI-R (Table 1). PCR product was digested with NheI-HF and XhoI restriction enzymes (New England Biolabs) and purified using a QIAquick PCR purification kit (Qiagen).

A construct containing the Δ1D2A sequence in a pCRII vector was used as a cloning template for construction of GLuc-Δ1D2A. The vector was digested with NheI-HF and XhoI restriction enzymes (New England Biolabs) and purified using a QIAquick PCR purification kit (Qiagen). Ligation of digested GLuc sequence into digested pCRII vector was performed using T4 DNA ligase (Roche). Creation of the GLuc-Δ1D2A chimera in the pCRII vector was confirmed by sequencing with T7 and GLuc-NS-NheI-R (Table 1), as described above.

For insertion of GLuc-Δ1D2A into pTarget plasmid PCR amplification was performed with pCRII GLuc-Δ1D2A as a template and using OneTaq 2× Master Mix with Standard Buffer (New England Biolabs) and primers AscI-Kzk-GLuc-F and 2A-XmaI-R (Table 1). Template pTarget GLuc was digested with AscI and XmaI restriction enzymes (New England Biolabs) and purified using QIAquick PCR purification kit (Qiagen). Ligation and confirmation by sequencing with T7 and Seq-R primers (Table 1) was performed as described above.

To produce SGLuc-Δ1D2A, site-directed mutagenesis was performed with primers SGLuc8990–MF and SGLuc8990–MR (Table 1), and using pTarget GLuc-Δ1D2A as a template as described above. Confirmation of mutagenesis was performed by sequencing with primers T7 and Seq-R (Table 1), as described above.

### Creation of Δ1D2A-GLuc/SGLuc chimeras

For construction of Δ1D2A-GLuc/SGLuc chimeras, nucleotide sequence encoding the Δ1D2A-SGLuc sequence was synthesized by GenScript and cloned into the pUC57kan vector. PCR amplification was performed using OneTaq 2× Master Mix with Standard Buffer (New England Biolabs) and primers AscI-Kzk-2A-F and GLuc-R-NotI (Table 1). Insertion into the pTarget vector (Promega) followed manufacturer’s instructions for T/A cloning. Confirmation of insertion was performed by sequencing with primers T7 and Seq-R as described above.

To construct the Δ1D2A-GLuc chimera the pTarget Δ1D2A-SGLuc construct was used as a template for site-directed mutagenesis using the GENEART Site-Directed Mutagenesis System (Invitrogen) with primers GLuc8990–MF and GLuc8990–MR (Table 1). Confirmation of mutation was performed by sequencing with primers T7 and Seq-R as described above.

### Creation of Δ1D2A-GLuc/SGLuc Δ1M chimeras

To construct Δ1D2A-GLuc/SGLuc Δ1M chimeras, pTarget Δ1D2A-GLuc and pTarget Δ1D2A-SGLuc template were used for site-directed mutagenesis using the GENEART Site-Directed Mutagenesis System (Invitrogen) with primers No Met GLuc-MF and No Met GLuc-MR (Table 1). Sequencing confirmation used primers T7 and Seq-R (Table 1), as described above.

### Creation of FMDV VLP producing constructs

Nucleotide sequence derived from FMDV O1 Manisa serotype and coding for the P1 polyprotein was synthesized by GenScript and cloned into a modified pTarget vector using BamHI-HF and NotI-HF restriction enzymes (New England Biolabs). For the P1-2A-3C construct, insertion of FMDV 3C was performed by using PCR amplification with primer NotI-3CLeb89-F and primer 3CLeb89-EcoRI-R (Table 1). For creation of the P1-2A-3C-Δ1D2A-SGLuc construct, insertion of 3C was performed by using PCR amplification with primer NotI-3CLeb89-F and 3CLeb89-ns-EcoRI-R (Table 1). The sequence for Δ1D2A-SGLuc was inserted 3′ of the 3C sequence by digestion of the template with EcoRI-HF and XmaI restriction enzymes (New England Biolabs). All ligations were performed as described above. Confirmation of insertion was performed by sequencing with primers NotI-3CLeb89-F, 3CLeb89-EcoRI-R, and Seq-R (Table 1), and analyzed as described above.

### Transfection and harvesting of HEK293-T cells

HEK293-T cells (ATCC® CRL-11268™) were maintained in 1× MEM media (Gibco) with 10% Fetal Bovine Serum defined (HyClone), 1× Antibiotic-Antimycotic (Gibco), 1× MEM-NEAA (Gibco), and 1× Glutamax (Gibco). At passage 58, HEK293-T cells were transfected with each of the six bicistronic chimeras, a negative control plasmid (Histone), or one of two positive control plasmids (GLuc and SGLuc) in a six-well plate using Lipofectamine 2000 (Invitrogen). After incubating for 24 h in a 37 °C CO_2_ incubator, media was removed and stored at 4 °C. Attached cells were removed from plates with 200 μl of 2× Luciferase Cell Lysis Buffer (Thermo Fisher Scientific) and stored at -70 °C.

### Luciferase assay

To adjust for differential expression of transgene among samples, harvested media was mixed with 4× NuPage LDS Sample Buffer (Invitrogen), heated at 97 °C for 10 min, and loaded into wells of a 10-well NuPage 4-12% Bis-Tris gels (Invitrogen). Gels were electrophoresed in 1× MES buffer (Invitrogen) at 200 V for 35 min. Samples were transferred onto membranes using the i-Blot system (Invitrogen). Membranes were incubated in 5% milk blocking buffer for 40 min and washed 3 times with 1× phosphate buffered saline (PBS)-Tween (PBS-T) buffer (EMD Millipore) for 5 min each. A 1:1000 dilution of rabbit polyclonal Antisera-GLuc (NanoLight Technologies) was used for primary antibody incubation with shaking at room temperature for 1 h. Membranes were washed three times with 1× PBS-T for 5 min each after primary antibody incubation. A 1:500 dilution of goat anti-rabbit-HRP secondary antibody (KPL) was applied and incubated with shaking at room temperature for 1 h, followed by three washes with 1× PBS-T for 5 min each. DAB staining was performed using SIGMAFAST 3,3′-Diaminobenzidine tablets (Sigma) dissolved in 15 μl of ddH_2_O for 1 h followed by de-staining with two rounds of washing with 1× PBS-T for 5 min. Volumes of media loaded onto the gel were adjusted until roughly equal loading was obtained for each sample as shown by similar staining intensity.

Luciferase activity was measured using a 96-well BioSystems Veritas luminometer (Turner Biosystems). For unadjusted samples, 20 μl of harvested media was used and readings taken with no delay after an injection of 25 μl of 100 μM water soluble coelenterazine solution (NanoLight Technologies). An integration time of 0.5 s was used for data collection both before and after injection of coelenterazine. Readings for pre-injection were used to establish a baseline of light emission at the time of injection and subtracted from the post-injection values during data analysis. Replicates were averaged together to give relative luciferase units per half second (RLU/0.5 s). Media from non-transfected HEK293-T cells was used to dilute samples as needed to maintain a constant volume.

To measure the effects of cell lysis buffers 10 μl harvested GLuc/SGLuc media was mixed with 90 μl of either cell culture media, 2× Luciferase Cell Lysis Buffer (LCLB, ThermoFisher), or Mammalian Protein Extraction Reagent (M-PER, ThermoFisher Scientific). LCLB and M-PER were selected as test buffers to represent a buffer that is supplied with luciferase reporter assays, LCLB, and a commonly used mammalian cell lysis buffer, M-PER. A total of 100 μl was used in each well with no delay after an injection of 25 μl of 50 μM water soluble coelenterazine solution (NanoLight Technologies). Readings were processed as above and used to calculate percentage of luciferase activity retained in added lysis buffer compared to cell culture media.

### Transfection of LFBK-αVβ6 cells

LFBK-αVβ6 cell line [20, 21], was grown in 1× DMEM media (Gibco) with 10% Fetal Bovine Serum defined (HyClone), 1× Antibiotic-Antimycotic (Gibco), 1× MEM-NEAA (Gibco), and 1× Glutamax (Gibco). Cells were transfected at passage 44 using Lipofectamine 2000 (Invitrogen). After incubating for 24 h in a 37 °C CO_2_ incubator, media from transfected cells were removed and used for luciferase assays as previously described while cells were harvested and used for immunoassays.

### IEM and IFA

To provide the volume of cells necessary for immunoelectron microscopy and for immunofluorescent assays (IFA), LFBK αVβ6 cells were grown and transfected in T-75 flasks. Immunoassays were performed using three different FMDV Mabs supernatants 6HC4, 12FE9, and F21 [22, 23]. The 6HC4 antibody does not react with FMDV Type O and was used as a negative control. Antibodies 12FE9 and F21 are reactive to FMDV type O VP1 and VP2 proteins respectively. For IEM, samples were fixed with a solution containing 4% paraformaldehyde (Electron Microscopy Sciences) with 1% periodate and 1% lysine in 0.1 M sodium cacodylate buffer (pH 7.4), embedded in 2% agarose, dehydrated to 70% ethanol, and embedded in medium grade LR White resin (Electron Microscopy Sciences). Ultrathin (80 nm) sections were cut on a Leica UC6 with a diamond knife, collected on formvar-carbon coated nickel grids (Electron Microscopy Sciences S), blocked (Nanoprobes), incubated with Mabs at a 1:10 dilution, and washed. Antibody binding was localized using Fab goat anti-mouse fragment conjugated to 1.4 nm nanogold (Nanoprobes) and enhanced with GoldEnhance EM (Nanoprobes). Labeled sections were contrasted with 2% uranyl acetate and lead citrate and imaged with a Hitachi 7600 at 80 kV with a digital camera (Advanced Microscopy Techniques).

For IFA, suspensions of transfected LFBK-αVβ6 cells were attached (CytoSpin) onto Superfrost Plus Gold Slides (EMS) and fixed with acetone:methanol at 4 °C for 10 min. Slides were blocked with 10% fetal bovine serum (FBS) and incubated with Mabs diluted 1:10 with 10% FBS. After washing 3 times with PBS (Gibco), bound antibodies were localized with a 1:500 dilution of Alexafluor 488 (Molecular Probes). Slides were washed 3 times with PBS and mounted with ProLong® Gold Antifade with DAPI (Molecular Probes). Fluorescent images were recorded with an Olympus Bx40.

## Abbreviations

FMDV: Foot-and-mouth disease virus
GLuc: *Gaussia* luciferase
GLuc/SGLuc: GLuc and SGLuc
IEM: Immunoelectron microscopy
RFUs: Relative fluorescent units
SGLuc: Super-luminescent *Gaussia* luciferase
VLP: Virus-like particle
Δ1D: Truncated 11 C-terminal amino acids of VP1
Δ1D2A: 2A translational interrupter sequence
